# Shorter handwashing at school than at home: perceived environmental factors

**DOI:** 10.3389/fpubh.2026.2001395

**Published:** 2026-10-21

**Authors:** Mami Osawa, Maki Kameoka, Nobuko Nakashima

**Affiliations:** 1 Division of Health Sciences, Program of Next-Generation Medical Technology, Graduate School of Medicine, Dentistry and Health Sciences, Niigata University, Niigata, Japan; 2 Sakeology Center, Niigata University, Niigata, Japan; 3 Professional School of Teacher Education, Niigata University, Niigata, Japan

**Keywords:** hand hygiene, handwashing, hygiene behavior, school environment, school toilets, schoolchildren

## Abstract

**Introduction:**

Handwashing after toilet use is an important hygiene practice, but the school environment in which children wash their hands has received comparatively little attention. This study examined perceived differences in handwashing duration between school and home and reasons for shorter handwashing at school among schoolchildren.

**Methods:**

A cross-sectional, anonymous, web-based questionnaire was administered to elementary and junior high school students in Niigata, Japan. Handwashing practices, perceived school–home differences in handwashing duration, reasons for shorter handwashing at school, and desired improvements to school toilets were assessed. Exploratory adenosine triphosphate, diphosphate, and monophosphate (A3) measurements of hands (*n* = 4) and restroom surfaces were conducted as complementary assessments.

**Results:**

Among 195 participants with available data, 43 (22.1%) reported shorter handwashing at school than at home. Among 153 participants with available handwashing-duration data and a definite school–home comparison, the shorter group reported a significantly shorter duration than the not-shorter group (median [interquartile range], 15.0 s [10.0–20.0] vs. 20.0 s [10.0–30.0]; *p* = 0.014). Concern about other people (33.3%) and short break times (28.6%) were the most frequently reported reasons for shorter handwashing. Cleanliness and hygiene was the most frequently requested improvement. In the exploratory hand A3 assessment, values decreased after handwashing and drying but remained above the manufacturer’s reference value in all four participants.

**Conclusion:**

Some schoolchildren perceived shorter handwashing at school than at home, most often for social and time-related reasons. These findings suggest that social, temporal, and physical school conditions merit attention alongside individual hygiene education.

## 1 Introduction

Handwashing is a fundamental public health practice for the prevention of infectious diseases, particularly diarrheal and acute respiratory infections (1). Schools are important settings for hand hygiene among children, and hand hygiene interventions in educational settings have been investigated for their potential to reduce infection-related illnesses and absenteeism (2). Handwashing after toilet use is one of the key times for hand hygiene recommended by the World Health Organization (WHO) and the United Nations Children’s Fund (UNICEF) in community settings, including schools (1). Appropriate hand hygiene depends not only on whether hands are washed but also on how handwashing is performed. WHO/UNICEF guidelines recommend handwashing with plain soap and water for sufficient time to enable complete coverage of both hands with soap and thorough rubbing (1). Thus, understanding how schoolchildren perform handwashing after toilet use, including the time they spend washing their hands, is relevant to supporting appropriate hand hygiene practices in schools.

Handwashing behavior at school may be influenced not only by individual knowledge and attitudes but also by the physical, temporal, and social environment in which handwashing takes place. Qualitative and observational studies in school settings have identified limited time, the accessibility and quality of handwashing facilities, the availability of water and soap, facility maintenance, and social influences as factors that may facilitate or hinder handwashing (3–5). School-based intervention studies further suggest that changes in the physical and organizational environment can influence opportunities for handwashing and hygiene practices, although improvements in facilities or hygiene programs do not necessarily translate directly into consistent handwashing behaviors (6, 7). In Japan, school water, sanitation, and hygiene (WASH) infrastructure is generally well established; nevertheless, school toilet environments have been identified as an area requiring improvement (8). Together, these findings indicate that handwashing at school should be considered within the broader context of the environment in which the behavior occurs.

Previous studies have examined handwashing durations and techniques among schoolchildren in observational and experimental settings (9–12). Children’s handwashing practices have also been investigated in school and home settings. In Japan, handwashing practices among elementary school children have been assessed across different situations at school and home (13). More recently, a study of children aged 8–10 years separately assessed hand hygiene practices at school and home and found that most children reported performing hand hygiene similarly in both settings (14). However, the handwashing duration was not directly compared between schools and homes in that study. Moreover, although previous studies have shown that handwashing at school is influenced by environmental and contextual factors, little is known about whether children themselves perceive the time they spend washing their hands to differ between school and home or, where handwashing is perceived to be shorter at school, what they consider to contribute to that difference.

Therefore, the present study aimed to examine handwashing practices after school toilet use among elementary and junior high school students, with a particular focus on perceived differences in handwashing duration between school and home, and the reasons for shorter handwashing at school. We also explored students’ perceptions of the improvements required in the school toilet environment. In addition, exploratory measurements of adenosine triphosphate, diphosphate, and monophosphate (A3) on hands and restroom surfaces were conducted to provide complementary objective observations of organic residues alongside the questionnaire-based assessment. By linking children’s own accounts of how long they wash their hands to the social, temporal, and physical conditions of the school, this study addresses an aspect of school hand hygiene that has received limited attention.

## 2 Materials and methods

### 2.1 Study design and participants

This cross-sectional study included an anonymous web-based questionnaire survey conducted between February and March 2023 among students attending Niigata University Affiliated Niigata Elementary School and Niigata University Affiliated Niigata Junior High School in Niigata, Japan. A total of 812 students were eligible for the survey, and 222 accessed the web-based questionnaire. No *a priori* sample size calculation was performed because this was an exploratory study, and all eligible students at the participating schools were invited to participate. Of these, 23 declined to participate and were excluded. One additional participant was excluded because of substantial missing data, resulting in 198 participants included in the final analysis. In addition to the questionnaire survey, exploratory A3 assessments of hands and restroom environmental surfaces were conducted as complementary assessments.

### 2.2 Questionnaire survey

The hand hygiene questionnaire was developed specifically for this study with reference to a previous questionnaire-based study of handwashing practices among Japanese elementary school children (13). An initial draft, including items concerning reasons for shorter handwashing at school, was prepared by the first author, and the questionnaire items and response options were subsequently reviewed and revised by all three authors. The questionnaire content was also shared with the participating schools before survey administration. The questionnaire content and response options were the same for elementary and junior high school students. To ensure age-appropriate readability, the use of kanji and hiragana in the Japanese questionnaire was adjusted according to school level. No separate formal pretesting was conducted specifically for junior high school students. The questionnaire was administered as an anonymous, self-administered web-based survey. Printed sheets containing the URL and QR code for the questionnaire were distributed to students through their homeroom teachers at the participating elementary and junior high schools. Students could complete the questionnaire either at school or at home. The researchers did not record whether teachers or parents were present when individual participants completed the questionnaire. The questionnaire collected information on participants’ demographic characteristics, handwashing practices after school toilet use, and perceptions of the school toilet environment. Demographic variables included school grade and sex. Hand hygiene-related items included the frequency, method, and duration of handwashing after school toilet use; handwashing duration at school compared with that at home; reasons for shorter handwashing at school; and hand-drying methods. The participants were also asked to provide free-text responses regarding the improvements they wanted to see in school toilets.

#### 2.2.1 Assessment of handwashing behavior

Handwashing frequency after school toilet use was assessed using a four-point ordinal response format: “always,” “usually,” “rarely,” and “never.” Handwashing methods were categorized as soap and water, alcohol-based hand sanitizer, water only, and no handwashing. Handwashing duration was self-reported in seconds. Participants were also asked to compare their handwashing duration at school with that at home using four response options: “shorter,” “no difference,” “longer,” and “do not know.” These questionnaire items were analyzed separately and were not combined across items to generate a composite score. For the analysis of shorter handwashing at school, participants who answered “shorter” were classified into the shorter group, whereas those who answered “no difference” or “longer” were classified into the not-shorter group. Participants who answered “do not know” were excluded from the analysis. Hand-drying methods were categorized as handkerchief, tissue, hair, clothing, or air drying.

#### 2.2.2 Reasons for shorter handwashing at school

All participants were asked about the reasons for shorter handwashing at school, and multiple responses were allowed; however, only the responses of participants who had reported shorter handwashing at school than at home were included in the analysis. The predefined response options were no specific reason, feeling unsettled, concern about other people, an unclean washing area, other people being in the restroom, or a short break. Participants could also select “other” and provide a free-text response. Free-text responses provided under “other” were reviewed and categorized according to their content, with additional categories created where appropriate; responses that could not be clearly classified into a specific category were retained as “other.”

#### 2.2.3 Requested improvements to school toilets

Participants were asked to provide a free-text response regarding the improvements they wanted to see in the school toilets. Categories were developed inductively from the content of the free-text responses by the first author (MO). The first author then coded all responses according to these categories. The second author (MK) independently coded the same responses using the category definitions without access to the first author’s coding results. The coding results of the two authors were compared, and any disagreements were resolved through discussion. Responses indicating that no improvement was needed (e.g., ‘nothing’) were treated separately and were not included in the categorization of specific improvement suggestions. When a response contained more than one distinct improvement, each was classified separately; therefore, a single participant could contribute to more than one category. Percentages for each improvement category were calculated using the 72 participants who provided at least one specific suggestion as the denominator.

### 2.3 Exploratory A3 assessments

Exploratory A3 assessments were performed using a Lumitester Smart luminometer and LuciPac A3 Surface swabs (Kikkoman Biochemifa Company, Tokyo, Japan). The A3 assay is a bioluminescence-based method for rapidly assessing organic residues on hand and environmental surfaces by detecting adenosine triphosphate (ATP) and its degradation products, adenosine diphosphate (ADP) and adenosine monophosphate (AMP), with results expressed in relative light units (RLU) (15). Higher RLU values indicate greater amounts of ATP-, ADP-, and AMP-containing residues on the sampled surface.

#### 2.3.1 Hand A3 assessments

Hand A3 assessments were conducted in four students (three boys and one girl) attending Niigata University Affiliated Niigata Junior High School. The assessments were performed in school restrooms normally used by the participants. Participants performed a simulated toilet use procedure in a toilet stall, mimicking their usual contact with surfaces such as the stall door and lock, toilet paper, and flush lever. The A3 values were measured for both hands after the simulated toilet-use procedure and again after handwashing and drying. Participants washed their hands using their usual handwashing method and duration, and subsequently dried them using their usual method. For A3 sampling, the palms, interdigital spaces, and fingertips were swabbed according to the manufacturer’s recommended hand sampling procedure. The A3 values were measured separately for the right and left hands and are expressed as RLU.

#### 2.3.2 Restroom environmental A3 assessments

Environmental A3 assessments were conducted in women’s restrooms at the Niigata University School of Health Sciences as a complementary exploratory assessment. This assessment was not intended to directly evaluate or represent the restroom environments of the participating schools. Toilet-related surfaces were sampled from 21 toilet stalls, including outer and inner toilet door knobs, toilet door surfaces, flush bars/levers, and toilet seats. The sink and sink shelves were sampled at 12 locations. One A3 measurement was obtained at each sampling location for each surface type. Surfaces were swabbed using LuciPac A3 Surface swabs, and A3 values were measured using Lumitester Smart and expressed as RLU.

### 2.4 Statistical analysis

Analyses were conducted using available data; therefore, the number of participants included in individual analyses varied because of missing responses. Handwashing duration is presented as the median and interquartile range (IQR), and categorical variables are presented as numbers and percentages. Participant characteristics and handwashing practices were compared between the shorter and not-shorter groups. Categorical variables were compared using the chi-square test or Fisher’s exact test, as appropriate, and handwashing duration was compared using the Mann–Whitney U test. Environmental A3 values are presented as medians, IQRs, and ranges. All statistical tests were two-sided, and statistical significance was set at *p* < 0.05. Statistical analyses were performed using IBM SPSS Statistics version 28.0.1 (IBM Corp., Armonk, NY, USA).

## 3 Results

### 3.1 Participant characteristics and hand hygiene practices

In total, 198 participants were included in the final analysis (Table 1). Among the 197 participants with available data on school level, 87 (44.2%) were elementary school students and 110 (55.8%) were junior high school students. Most participants (89.8%) reported that they always washed their hands after school toilet use, and 75.1% reported washing their hands with soap and water. The median self-reported handwashing duration was 20.0 s (IQR, 10.0–30.0 s). Among the 195 participants with available data on handwashing duration at school compared with that at home, 43 (22.1%) reported shorter handwashing at school, 105 (53.8%) reported no difference, and 27 (13.8%) reported longer handwashing. A handkerchief was the most commonly reported hand-drying method (70.8%), followed by letting hands air-dry (19.5%).

**Table 1:** Participant characteristics and hand hygiene practices.

Characteristic	Overall (*n* = 198)
Sex, *n* (%) (*n* = 197)	
Boys	83 (42.1)
Girls	114 (57.9)
School level, *n* (%) (*n* = 197)	
Elementary school	87 (44.2)
Junior high school	110 (55.8)
Handwashing after school toilet use, *n* (%) (*n* = 197)	
Always	177 (89.8)
Usually	16 (8.1)
Rarely	3 (1.5)
Never	1 (0.5)
Handwashing method, *n* (%) (*n* = 197)	
Soap and water	148 (75.1)
Alcohol-based hand sanitizer	5 (2.5)
Water only	43 (21.8)
No handwashing	1 (0.5)
Handwashing duration, s, median [IQR] (*n* = 170)	20.0 [10.0–30.0]
Handwashing duration at school compared with home, *n* (%) (*n* = 195)	
Shorter	43 (22.1)
No difference	105 (53.8)
Longer	27 (13.8)
Do not know	20 (10.3)
Hand-drying method, *n* (%) (*n* = 195)	
Handkerchief	138 (70.8)
Tissue	2 (1.0)
Hair	6 (3.1)
Clothing	11 (5.6)
Letting hands air-dry	38 (19.5)

Values are presented as *n* (%) unless indicated otherwise. Percentages were calculated using the number of available responses for each item as the denominator. Handwashing duration was summarized for participants who provided a single numeric response.

### 3.2 Characteristics and reasons for shorter handwashing at school

Of the 195 participants with available data on handwashing duration at school compared with that at home, 175 provided a definite comparison; of these, 43 were classified into the shorter group and 132 into the not-shorter group (Table 2). Among the 153 participants with available handwashing-duration data, the shorter group reported a significantly shorter handwashing duration than the not-shorter group (median [IQR], 15.0 [10.0–20.0] vs. 20.0 [10.0–30.0] s; *p* = 0.014). No significant differences were observed between the groups in sex, school level, handwashing frequency, handwashing method, or hand-drying method.

**Table 2:** Characteristics according to shorter handwashing at school than at home.

Characteristic	Not shorter (*n* = 132)	Shorter (*n* = 43)	*p*
Sex, *n* (%) (*n* = 131 and 43)			0.667
Boys	53 (40.5)	19 (44.2)	
Girls	78 (59.5)	24 (55.8)	
School level, *n* (%) (*n* = 131 and 43)			0.652
Elementary school	60 (45.8)	18 (41.9)	
Junior high school	71 (54.2)	25 (58.1)	
Handwashing after school toilet use, *n* (%) (*n* = 132 and 43)			0.567
Always	117 (88.6)	40 (93.0)	
Less than always	15 (11.4)	3 (7.0)	
Handwashing method, *n* (%) (*n* = 132 and 43)			0.376
Soap and water	101 (76.5)	30 (69.8)	
Other methods	31 (23.5)	13 (30.2)	
Handwashing duration, s, median [IQR] (*n* = 114 and 39)	20.0 [10.0–30.0]	15.0 [10.0–20.0]	0.014^*^
Hand-drying method, *n* (%) (*n* = 132 and 42)			0.392
Handkerchief	97 (73.5)	28 (66.7)	
Other methods	35 (26.5)	14 (33.3)	

Values are presented as n (%) unless indicated otherwise. Percentages were calculated using the available data for each variable as the denominator. “Less than always” included “usually,” “rarely,” and “never.” “Other methods” for handwashing included alcohol-based hand sanitizer, water only, and no handwashing; “Other methods” for hand drying included tissue, hair, clothing, and air drying. Sample sizes are presented in the order of the not-shorter and shorter groups. *p* values were calculated using the chi-square test, except for handwashing after school toilet use (Fisher’s exact test) and handwashing duration (Mann–Whitney *U* test). *p < 0.05.

Among participants in the shorter group who provided responses regarding reasons for shorter handwashing at school (*n* = 42), the most frequently reported reason was concern about other people (33.3%), followed by short break times (28.6%) and no specific reason (23.8%) (Figure 1). An unclean washing area was reported by 14.3% of participants, while cold water and the presence of other people in the restroom were each reported by 9.5%.

**Figure 1:**
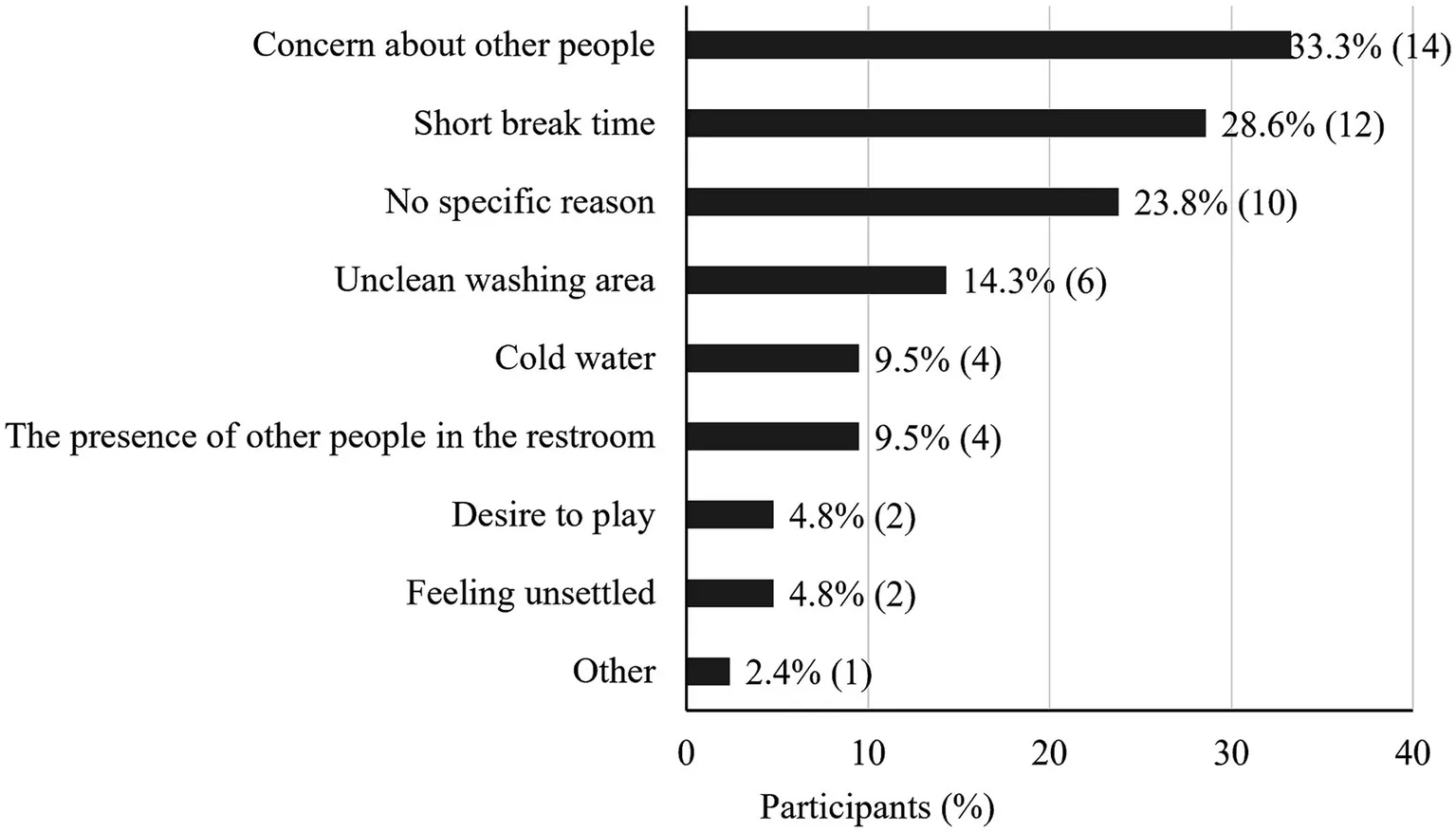
Reasons for shorter handwashing at school reported by students who perceived their handwashing to be shorter at school than at home. Data are presented for the 42 of 43 students who provided at least one response. Multiple responses were permitted; therefore, percentages do not sum to 100%.

### 3.3 Requested improvements to school toilets

Among the 198 participants, 72 provided at least one specific suggestion for improving school toilets, while 46 indicated that no improvement was needed. Among the 72 participants who provided at least one specific suggestion, the most frequently reported category was cleanliness and hygiene (29.2%), followed by other toilet facilities and equipment (18.1%), user behavior and social environment (16.7%), water temperature (13.9%), and privacy (12.5%) (Figure 2). Other categories included toilet availability and capacity (8.3%), crowding and space (8.3%), and handwashing facilities and supplies (6.9%). Representative responses within each category are presented in Supplementary Table S1.

**Figure 2:**
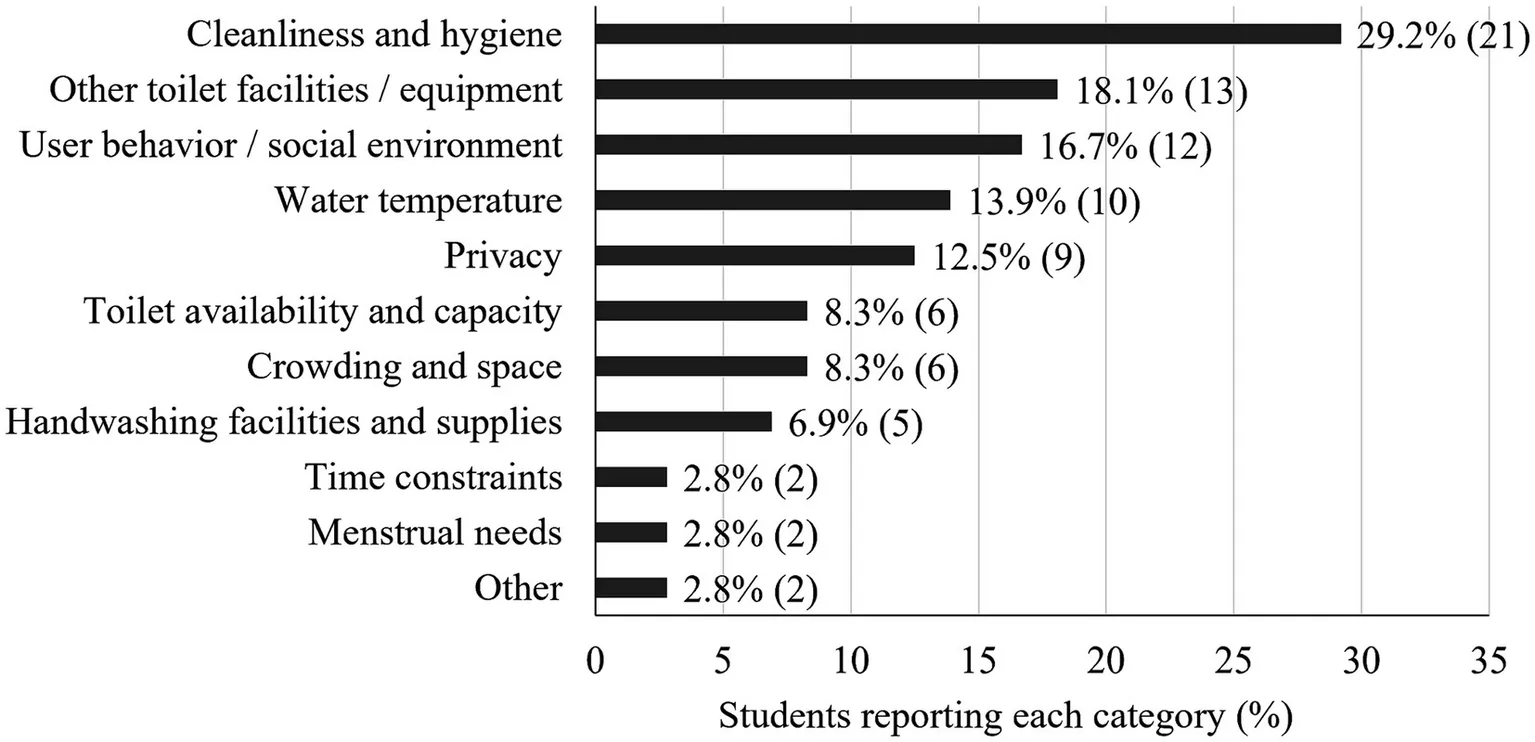
Requested improvements to school toilets reported by students. Percentages were calculated for the 72 students who provided at least one specific suggestion for improving school toilets. Participants could contribute to more than one category when their responses contained multiple distinct improvements; therefore, percentages may sum to more than 100%.

### 3.4 Exploratory A3 assessments

All four participants washed their hands for 10–15 s using their usual handwashing method. A3 values generally decreased after handwashing and drying compared with those measured after the simulated toilet-use procedure, although the magnitude of change varied among participants. After handwashing and drying, the A3 values for both hands of all four participants remained above the manufacturer’s reference value of 2,000 RLU (Supplementary Figure S1).

The environmental A3 values varied across restroom surfaces (Supplementary Figure S2 and Supplementary Table S2). The highest median A3 value was observed on sink shelves (16,947 RLU [IQR, 11,303–72,567]), followed by toilet seats (9,835 RLU [IQR, 7,343–12,520]) and sinks (7,286 RLU [IQR, 5,164–15,028]). The lowest median A3 value was observed on flush bars/levers (1,257 RLU [IQR, 951–1,505]).

## 4 Discussion

In this study, most students reported that they routinely washed their hands after using school toilets; however, approximately one in five reported that their handwashing was shorter at school than at home (Table 1). Students who reported shorter handwashing at school also reported a significantly shorter handwashing duration than those who did not (Table 2). Among students reporting shorter handwashing at school, concern about other people was the most frequently reported reason, followed by short break times (Figure 1). These findings suggest that perceived differences in handwashing duration between school and home may relate not only to individual handwashing practices but also to the social and temporal context of the school environment.

Current WHO/UNICEF guidelines recommend handwashing with soap and water for a sufficient time to allow complete coverage and thorough rubbing of both hands (1). Although duration alone does not determine the effectiveness of hand hygiene, sufficient time is an integral component of the handwashing process. Previous studies have examined handwashing duration and techniques among schoolchildren (9–12). However, relatively little attention has been paid to whether children perceive their handwashing duration to differ across everyday settings, particularly between school and home. The present findings, therefore, add to previous research by showing that a subset of students perceived their handwashing to be shorter at school than at home and that this perception was accompanied by a significantly shorter self-reported handwashing duration.

Previous studies have shown that handwashing behavior among schoolchildren can be influenced by the social and temporal contexts in which it occurs. In Kenyan primary schools, students were more likely to clean their hands after toilet use when other students were present at a handwashing station (16). Qualitative research has also shown that social environment, including interactions with peers and teachers, can facilitate or hinder handwashing practices in schools (4). These findings suggest that the presence and behavior of other people may shape handwashing behavior, although the direction and nature of this influence may vary according to the context. In the present study, concern about other people was the most frequently reported reason for shorter handwashing at school. This finding raises the possibility that the social environment surrounding school toilets and handwashing facilities may influence not only whether children wash their hands, but also the time they spend doing so. However, the questionnaire did not assess the specific nature of this concern. Further studies are needed to determine whether this reflects being observed by peers, crowding, embarrassment, pressure to leave the washing area, or other social factors.

Time constraints may be another important contextual factor. Studies in UK primary schools have identified insufficient time, rushing, and time lost while waiting to use handwashing facilities as barriers to thorough handwashing (3, 17). Studies from other school settings have similarly identified organizational and environmental constraints, including access to facilities, availability of water and soap, and opportunities to perform handwashing, as relevant to children’s handwashing practices (5–7). The frequent reporting of short break times in the present study is therefore consistent with previous evidence that handwashing occurs within the practical constraints imposed by the school setting. Importantly, however, the present study assessed students’ perceived reasons rather than objectively measuring the break duration or time available for handwashing; thus, these findings should be read as perceived barriers rather than as causal effects of school scheduling.

Students also identified a broad range of desired improvements in the school toilet environment, with cleanliness and hygiene the most frequently reported category, followed by other toilet facilities and equipment, user behavior and social environment, water temperature, and privacy (Figure 2; Supplementary Table S1). Previous studies have similarly demonstrated that children’s experiences and use of school toilets are shaped by multiple dimensions of the toilet environment, including cleanliness, privacy, accessibility, facility conditions, hygiene supplies, and peer or social factors (18–25). In particular, Shao et al. identified cleanliness as an important factor in primary school students’ toilet use (21), whereas studies in other populations of schoolchildren have documented dissatisfaction with, or avoidance of, school toilets because of environmental and social conditions (22–25). Studies conducted in Japanese elementary and junior high schools have also highlighted the spatial and social aspects of school toilets from children’s perspectives (26), while a broader review of WASH conditions in Japanese elementary schools indicates that despite the generally well-established water and sanitation infrastructure, the need for improvements in school toilet environments remains (8). In addition, inadequate provision of warm water, soap, and hand-drying facilities has been documented in school handwashing facilities (27). School-based hygiene studies have also considered toilet cleanliness, handwashing facilities, and hygiene supplies as components of the school hygiene environment (6, 28).

The convergence between these previous findings and the improvements requested by students in the present study underscores the value of considering children’s perceptions when evaluating school toilet environments. However, the requested improvements assessed in this study were obtained separately from the questions on reasons for shorter handwashing. They should therefore not be interpreted as factors that cause shorter handwashing at school. Rather, they provide complementary information on the aspects of the broader toilet environment that students perceive as needing improvement. Future studies could directly examine whether specific environmental characteristics, such as cleanliness, privacy, crowding, water temperature, availability of supplies, and the configuration of handwashing facilities, are associated with objectively measured handwashing duration or quality. Combining children’s perspectives with objective assessments may help identify school toilet environments that are acceptable to students and support appropriate hand hygiene practices.

Bioluminescence-based ATP measurements have been used to assess residues on children’s hands and restroom surfaces, and A3 testing extends this approach by detecting ATP together with ADP and AMP (15, 29–31). In the exploratory hand A3 assessment, all four students washed their hands using their usual methods for 10–15 s, which fell within the IQR of the self-reported handwashing duration in the shorter group (median [IQR], 15.0 s [10.0–20.0]). Although A3 values decreased after handwashing and drying, the values for both hands of all four students remained above the manufacturer’s reference value of 2,000 RLU (29) (Supplementary Figure S1). Given the very small number of participants, these measurements cannot be interpreted as evidence of inadequate handwashing or of microbial contamination, nor do they establish any relationship between handwashing duration and post-handwashing A3 values; they are reported only as exploratory observations accompanying handwashing within the range of durations reported in the questionnaire survey. The environmental A3 values also differed across restroom surfaces, with the highest median value observed on sink shelves, followed by toilet seats and sinks (Supplementary Figure S2 and Supplementary Table S2). Together, these exploratory measurements complement the questionnaire data and are consistent with the broader point that handwashing practices and the environment in which they occur are best considered together.

This study has several strengths, including its focus on perceived differences in handwashing duration between school and home, its assessment of students’ own reasons for shorter handwashing at school, and its use of both quantitative and free-text data. It also has some limitations. First, the cross-sectional design precludes causal inference, and handwashing practices and duration were self-reported and may therefore be subject to recall and social desirability bias. Similarly, findings regarding requested improvements to school toilets were based only on participants who provided specific suggestions and may not represent the views of the entire study population. Second, the study was conducted at two university-affiliated schools in one region of Japan, which may limit the generalizability of the findings. In addition, only 198 of the 812 eligible students were included in the final analysis, and participation was voluntary. Therefore, non-response or selection bias cannot be excluded, as students with greater interest in hand hygiene or school toilet environments may have been more likely to participate. Third, the hand hygiene questionnaire was developed specifically for this study and did not undergo formal psychometric validation; therefore, the validity and reliability of the questionnaire items remain to be established. Fourth, the questionnaire did not assess in detail what students meant by “concern about other people,” limiting interpretation of the specific social factors underlying this response. Finally, the hand A3 assessment was exploratory and included only four students, so these measurements should be regarded as illustrative rather than as findings in their own right. Larger and more diverse studies are needed to confirm the questionnaire findings and to examine the factors related to shorter handwashing at school. Despite these limitations, the present findings indicate that some students perceive their handwashing to be shorter at school than at home and that concern about other people and limited break times are the reasons reported most often. These results suggest that school health and hygiene promotion may need to address the social, temporal, and physical conditions under which children wash their hands, alongside individual hygiene education.

## Data Availability

The datasets presented in this article are not readily available because in accordance with the approved study protocol, the data are not permitted to be shared with other institutions. Further inquiries can be directed to the corresponding author. Requests to access the datasets should be directed to Mami Osawa, oswmami@clg.niigata-u.ac.jp.
