# Supplementary material for: Shorter handwashing at school than at home: perceived environmental factors
Source: Front Public Health. 2026 Oct 21;14:2001395. doi: 10.3389/fpubh.2026.2001395 (PMC13733523; doi:10.3389/fpubh.2026.2001395)
Supplement: Supplementary file 1 [file Data_Sheet_1.docx]

Supplementary Material

# Supplementary Table S1

Supplementary Table S1. Improvements to school toilets requested by students

Percentages were calculated for the 72 students who provided at least one specific suggestion for improving school toilets. A single response could be assigned to more than one category when it contained multiple distinct concepts; therefore, the percentages did not sum to 100%.

# Supplementary Figure S1

# A. Right hand

# B. Left hand

Supplementary Figure S1. Individual changes in hand A3 values after simulated toilet use and subsequent hand washing and drying.

A3 values were measured separately for the (A) right and (B) left hands of the four participants. Individual lines represent the participants. A3 values generally decreased after handwashing and drying, although the magnitude of change varied among participants. The y-axis is presented on a logarithmic scale because of the wide range of A3 values across participants.

# Supplementary Figure S2

Supplementary Figure S2. Distribution of A3 values across environmental surfaces in university restrooms

A3 values were measured for seven types of environmental surfaces in women’s university restrooms. Individual data points and box-and-whisker plots are shown. A3 values were log-transformed for visualization because of their wide and right-skewed distributions.

# Supplementary Table S2

Supplementary Table S2. A3 values measured on environmental surfaces in university restrooms.

Data are presented as median [interquartile range] and range. A3 values are expressed as relative light units (RLU).
